# Being left-behind, mental disorder, and elderly suicide in rural China: a case–control psychological autopsy study

**DOI:** 10.1017/S003329171800106X

**Published:** 2018-04-25

**Authors:** Liang Zhou, Guojun Wang, Cunxian Jia, Zhenyu Ma

**Affiliations:** 1 The Affiliated Brain Hospital of Guangzhou Medical University (Guangzhou Huiai Hospital), Guangzhou, China; 2 Xiangya School of Public Health, Central South University, Changsha, China; 3 School of Public Health, Shandong University, Jinan, China; 4 School of Public Health, Guangxi Medical University, Nanning, China

**Keywords:** Diagnosis, risk factors, suicide

## Abstract

**Background:**

Suicide rate among rural elderly is the highest among all age groups in China, yet little is known about the suicide risks in this rapidly growing vulnerable population.

**Methods:**

This matched case–control psychological autopsy study was conducted during June 2014 to September 2015. Consecutive samples of suicides aged 60 or above were identified in three provinces (Shandong, Hunan, and Guangxi) in China. Living comparisons were 1:1 matched with the suicides in age (±3 years old), gender, and living location. Risk factors included demographic characteristics, being left-behind, mental disorder, depressive symptoms, stressful life events, and social support.

**Results:**

A total of 242 suicides and 242 comparisons were enrolled: 135 (55.8%) were male, mean (s.d.) age was 74 (8) years. The most frequently used suicide means were pesticides (125, 51.7%) and hanging (95, 39.3%). Independent risks of suicide included unstable marital status [odds ratio (OR) 4.19, 95% confidence interval (CI) 1.61–10.92], unemployed (compared with employed, OR 4.43, 95% CI 1.09–17.95), depressive symptoms (OR 1.34, 95% CI 1.21–1.48), and mental disorder (OR 6.28, 95% CI 1.75–22.54). Structural equation model indicated that the association between being left-behind and suicide was mediated by mental disorder, depressive symptoms, stressful life events, and social support.

**Conclusions:**

Unstable marital status, unemployed, depressive symptoms, and mental disorder are independent risk factors for suicide in rural elderly. Being left-behind can elevate the suicide risk through increasing life stresses, depressive symptoms, mental disorder, and decreasing social support. Elderly suicide may be prevented by restricting pesticides, training rural physicians, treating mental disorders, mitigating life stress, and enhancing social connection.

## Introduction

Suicide is a major public health and mental health challenge. In China, the high suicide rates and unique suicide patterns observed in the 1990s (Phillips *et al.* 2002*a*) have been changed in recent years. Based on the Global Burden of Disease study, suicide rate in China has decreased from 20 per 100 000 in 1990 to 12 per 100 000 in 2010 (Yang *et al.* 2013). Using different data resources and methods, researchers drew similar conclusions (Wang *et al.* 2014; Zhang *et al.* 2014; Liu *et al.* 2015). The pattern of suicide has also changed dramatically. Suicide rates among male have exceeded those among female; the peak of suicide in rural young women has gradually vanished; the rural/urban ratio of suicide rates has been significantly reduced, while suicide rates remain the highest in older adults (Wang *et al.* 2014; Zhang *et al.* 2014; Liu *et al.* 2015). During 2013–2014, it was estimated that the average suicide rate among elderly (65 or above) was 34.5 per 100 000, 6.5-fold higher than the rate of population under 65, and rural elderly were more likely to commit suicide than urban elderly (incidence rate ratio = 1.83) (Zhong *et al.* 2016). Researches indicated that urbanization and economic development might be the main reasons for these changes (Liu *et al.* 2015; Sha *et al.* 2017). However, a decomposing study showed that the positive impact of urbanization on suicide rate had diminished recently, and suicide among older adults might be elevated as China is facing slower economic growth and rapid aging (Sha *et al.* 2017).

Remarkable demographic transition has happened in China in the past three decades. China stepped into an aging society in 1999, and it has 143.9 million older adults now (65 or above) or 10.5% of the whole population (National Bureau of Statistics of China, 2017). Approximately 250 million rural residents (40% of the whole rural population) move to urban areas each year, mostly young and middle-aged migrant workers. Therefore, a large number of rural elderly are left-behind. Traditionally Chinese elderly are taken care of by their children, but this has become difficult because of urbanization, domestic migration, and the deconstruction of extended families. Concerns about the impact of urbanization on rural elderly has raised not only in China but also in other developing and urbanizing countries.

Previous studies on the mental health status of left-behind elderly showed inconsistent results. In a study in rural China, authors reported that the migration of rural workforce had significantly degraded the welfare of the left-behind elderly (He and Ye, 2014). Several quantitative studies showed that 41–56% Chinese rural older adults were empty-nester or left-behind, and it was correlated to worse mental health outcomes including loneliness, depression, and anxiety (Wang *et al.* 2013, 2017). On the contrary, researches in Thailand and Indonesia reported less negative effects or even positive effects: children working in urban areas can provide better material support for their elderly parents; negative impacts of migration on social support are attenuated by support from kin and neighbors, the advent of communication technologies, and the improvements of transportation; pre-existing advantages of families that send migrants (Kreager, 2006; Knodel and Saengtienchai, 2007; Abas *et al.* 2009).

Living arrangements, rather than being left-behind, have been examined in suicide risks studies. Living alone has been shown to be a risk factor of elderly suicide in a study in Australia (De Leo *et al.* 2013), but the association was not found in another study in Sweden (Rubenowitz *et al.* 2001). A study in Hong Kong found that living with children was a protective factor for both suicide attempt and completed suicide in older adults (Chiu *et al.* 2004). However, living arrangements might be less relevant in rural areas. It is common that elderly live alone while they are taken care of by their adult children who live nearby (i.e. in the same village). Thus, it is important to examine directly whether being left-behind is associated with elderly suicide, and if yes, what is the mechanism underlying the association.

To understand the characteristics and risk factors of suicide in this population is of key importance for suicide prevention. However, little is known about suicide among older adults in China. Using data from the Integrated National Mortality Surveillance System, Zhong *et al.* reported suicide rates and geographical distribution of Chinese older adults from 2013 to 2014 (Zhong *et al.* 2016). A review summarized characteristics of elderly suicides based on several reginal studies in China (Li *et al.* 2009). There have been two large-scale psychological autopsy studies in mainland China, the earlier one focused on general population was conducted in 2000 by Phillips *et al.* (Phillips *et al.* 2002*b*). The authors further described the characteristics of 304 suicides older than 55 years in the sample (Zhou *et al.* 2004; Wang *et al.* 2007). While providing important data about suicide in older adults in China, information from these studies is outdated, and more importantly, the value is limited because of the lack of comparisons. The later psychological autopsy study focused on younger suicides aged 15–34 (Zhang *et al.* 2010). Other studies on the risk factors of suicide in older adults used suicide attempt (Zhang *et al.* 2017*b*) or suicide ideation (Zhang *et al.* 2017*a*) as main outcomes.

Psychological autopsy is a widely used method to explore the risk factors of completed suicide (Conwell *et al.* 1996; De Leo *et al.* 2013) and has been validated previously in China (Zhang *et al.* 2003). Using 1:1 matched case–control design, this psychological autopsy study aims at examining the relationship of demographic characteristics including being left-behind, mental disorder, depressive symptoms, stressful life events, social support, and completed suicide. More specifically, we hypothesize that being left-behind elevates the risk of suicide, and mental disorder, depressive symptoms, life stress, and social support mediate the relationship between being left-behind and suicide. To the best of our knowledge, this is the first national psychological autopsy study focused on the risk factors of suicide in rural older adults, a rapidly growing vulnerable population in contemporary China.

## Material and methods

### Sample and sampling

Multi-stage stratified cluster sampling method was used to select the research sites. In the first stage, based on the GDP per capita of 31 provinces in mainland China, Shandong, Hunan, and Guangxi were chosen from the top 10, 11–20, and 21–31 provinces, respectively. Counties in these provinces were stratified into three strata based on average income. One county in each stratum in Shandong and Hunan provinces and two counties in each stratum in Guangxi province (because the population size of county in Guangxi was smaller) were randomly selected. Therefore, 12 counties from three provinces were chosen.

We relied on the death certification system in each county to consecutively collect completed suicides aged 60 or above. All village doctors and local public health professionals involved in death certification were trained briefly and were asked to report all elderly suicide death to local Center for Disease Control and Prevention (CDCs). The manner of death was finally determined by trained investigators after all available and relevant information was collected.

Living comparisons were 1:1 matched with the suicide case in age (±3 years old), gender, and living location. Whenever a suicide case was identified, the investigators would list and numerate all older adults that matched in age and gender in the same village. Then one living comparison was randomly selected from the list using a computer program. In a few cases when there were no appropriate living comparisons available, the investigators expanded the search to the nearest villages.

### Procedures of interview

This study was conducted during June 2014 to September 2015. Interviewers from the three research sites were trained intensively for 10 days on the determination of manner of death, method of psychological autopsy, interview skills, and administration of study instruments.

Interviews with informants of suicide victims were scheduled 2–6 months after death, while interviews with informants of living comparisons were conducted as soon as the participants and their informants were identified. Two informants for each suicide victim and living comparison were identified: generally, the first informant was one next-to-kin who lived with the suicide victim or living comparison, and the second informant was always a friend, a neighbor, or a remote relative. Each informant was interviewed separately by one trained interviewer. The average interview time was 90 min.

The study was approved by the IRB of the Central South University, Shandong University, and Guangxi Medical University. The aim and procedure of the research were explained to all participants. Written informed consent must be obtained before interviews were conducted.

### Measures

#### Demographic characteristics

Demographic characteristics including age, gender, marital status, family income, school education, living arrangement, and pesticides stored at home were collected. All adult children of each elderly in both groups were listed, and the living location and frequency of visiting their parents of each adult child were recorded. Being left-behind was defined as during the last 12 months prior to death (for suicides) or investigation (for living comparisons), all adult children had lived out of the original township for at least 10 months, and had visited their parents no more than twice.

#### Suicide behavior and help-seeking behavior before suicide

Information about the time, location, and method of suicide was collected. Suicide intent was assessed using the eight-item Beck's Suicide Intent Scale (SIS-8). We also asked if the suicide victim had sought for help from a doctor (and if yes, what kind of doctor) in the last month prior to suicide.

#### Mental disorder

The Chinese version of the Structured Clinical Interview for the Diagnostic and Statistical Manual of Mental Disorders, Fourth Edition (DSM-IV; SCID) was used to generate current diagnoses of mental disorder. Diagnoses were made by psychiatrists in consensus meetings in which all information including SCID interview from both informants and previous medical records was presented. Four categories of diagnoses were included: mood disorders, schizophrenia and other psychotic disorders, alcohol dependence, and anxiety disorders. Because complex general medical conditions and medications were common in older adults, diagnoses including major depressive episode and psychotic disorder not otherwise specified were used if appropriate. Diagnoses of personality disorders or uncommon disorders in Chinese rural elderly (i.e. mental disorder onset in childhood and adolescence, eating disorder, illicit drug abuse, etc.) were not included. Multiple diagnoses were made if appropriate.

#### Depressive symptom, social support, and stressful life events

Geriatric depression scale (GDS) was used to assess the depressive symptoms in the last week before death/investigation. The instrument composed of 30 items (possible scores range from 0 to 30), and a higher score indicates severer depressive symptom (Chan, 1996). GDS has been validated in rural Chinese older adults in a previous study (He *et al.* 2008). The severity of depressive symptom can be categorized into three groups based on the GDS score: no or mild depression 0–10, moderate depression 11–20, and severe depression >20. Social support in the last week before death/investigation was measured by the 23-item Duke Social Support Index (DSSI, possible scores range from 11 to 45), and a higher score indicates higher social support. DSSI has been used in a previous psychological autopsy study in China and showed satisfactory reliability and validity (Zhang *et al.* 2003). Stressful life events in the last 12 months before death or investigation were measured by the Life Events Scale for the Elderly (LESE), which was developed specifically for older adults in China and covered 46 life events (Xiao and Xu, 2008).

### Integration of information from different sources

Answers from the two informants may differ, therefore need to be integrated. For the demographic characteristics and suicide behavior, we relied on the information provided by the first informant. For each item of GDS, DSSI, and LESE, answers that were hypothetically correlated to elevated suicide risk were used. The rationale for this practice is that a targeting behavior may exist when one of the two informants has observed it. For instance, positive answer of an item of GDS was used when one of the two informants reported positive; similarly, higher scores of LESE and lower scores of DSSI were used.

### Statistical method

Descriptive analysis, χ^2^ tests, *t* tests, and rank-order tests were used to describe and compare the demographic characteristics, mental disorder, depressive symptoms, life events, and social support of suicides and comparisons. Marital status was dichotomized into stable and unstable: the former included currently married and living together, the latter included never married, divorced, widowed, and separated. Family annual income was categorized into three groups based on the 33 and 66 percentiles: <3600, 3600–10 000, and >10 000. Adjusted odds ratios (ORs) and 95% confidence intervals (CIs) derived from the conditioned multivariable logistic regression indicated the associations between risk factors and suicide: social support was dichotomized into two groups based on the median score of DSSI; stressful life events was categorized into three groups based on the 33 and 66 percentiles; the score of GDS was used as a continuous variable to indicate the severity of depressive symptoms. Backward stepwise (likelihood ratio) method was used in the logistic regression. All these analyses were conducted using SPSS 24.0 for Windows. All reported *p* values were two-sided, and *p* values <0.05 were considered statistically significant.

The theoretical model was estimated with structural equation model. We speculated that being left-behind did not cause suicide directly, but elevated the suicide risk through its effects on life events, depressive symptoms, mental disorder, and social support. Life events not only had a direct effect on suicide, but also had an indirect effect through decreasing social support and increasing depressive symptoms and mental disorder. Depressive symptoms, mental disorder, and social support had direct effects on suicide and were correlated with each other. The χ^2^ test, goodness-of-fit index (GFI), adjusted goodness-of-fit (AGFI), Tracker–Lewis index (TLI), and root mean square error of approximation (RMSEA) were used to estimate the GIF of the theoretical model. These analyses were performed using AMOS 21.0 for Windows.

## Results

### Demographic characteristics, depressive symptoms, stressful life events, and social support of suicides and comparisons

A total of 242 suicide victims and 242 living comparisons were enrolled in this study. As shown in Table 1, 135 (55.8%) of them were male, and the mean (s.d.) age was 74 (8) years old. Compared with living comparison group, suicides were more likely to have unstable marital status, to be unemployed and be left-behind, and to live alone. No statistically significant difference was found in education, income, and storing pesticides at home between suicides and comparisons.

**Table 1.** Demographic characteristics, depressive symptoms, stressful life events, and social support of 242 completed suicides and 242 living comparisons

Demographic characteristics	Completed suicides	Living comparisons	χ^2^/*t*/*Z*	*p*
Gender (male, %)	135 (55.8%)	135 (55.8%)	–	–
Age (mean ± s.d.)	74.4 ± 8.2	74.1 ± 8.2	0.511	0.610
Education				
Below primary school	111 (45.9%)	96 (39.7%)	1.920	0.383
Primary school	105 (43.4%)	116 (47.9%)		
Above primary school	26 (10.7%)	30 (12.4%)		
Marital status				
Stable marital status	122 (50.4%)	170 (70.2%)	19.890	<0.001
Unstable marital status	120 (49.6%)	72 (29.8%)		
Employment				
Employed	40 (16.5%)	59 (24.4%)	7.837	0.021
Unemployed	195 (80.6%)	169 (69.8%)		
Retired	7 (2.9%)	14 (5.8%)		
Family annual income (RMB)				
<3600	88 (36.4%)	74 (30.6%)	1.865	0.394
3600–10 000	88 (36.4)	98 (40.5%)		
>10 000	66 (27.3%)	70 (28.9%)		
Living alone				
Yes	64 (26.4%)	35 (14.5%)	10.679	0.002
No	178 (73.6%)	207 (85.5%)		
Being left-behind				
Yes	41 (16.9%)	25 (10.3%)	4.491	0.034
No	201 (83.1%)	217 (89.7%)		
Pesticides stored at home				
Yes	127 (52.5%)	132 (54.5%)	0.208	0.715
No	115 (47.5%)	110 (45.5%)		
Depressive symptoms				
0–10	15 (6.2%)	156 (64.5%)	227.974	<0.001
11–20	71 (29.3%)	69 (28.5%)		
>20	156 (64.5%)	17 (7.0%)		
Number of stressful life events (median, interquartile ranges)	6 (4–8)	3.5 (2–7)	5.907	<0.001
Social support (mean ± s.d.)	15.4 ± 3.1	19.5 ± 3.1	−14.629	<0.001

Compared with living comparisons, suicides had significantly severer depressive symptoms, more stressful life events, and less social support (all *p* values <0.001). Most suicides (227, 93.8%) and 86 (35.5%) comparisons had at least moderate depressive symptoms. The median (interquartile ranges) of number of stressful life events in suicides and comparisons were 6 (4–8) and 3.5 (2–7), respectively. The three most common life stresses in suicides in the last year before death were suffer from chronic disease (191, 78.9%), hospitalization (132, 54.5%), and death of spouse (77, 31.8%).

### Suicide behavior and help-seeking behavior among suicides

The most frequently used suicide means in our sample were pesticides (125, 51.7%), followed by hanging (95, 39.3%), drowning (nine, 3.7%), and poisons other than pesticides (eight, 3.3%). Most suicides (212, 87.6%) happened in homes. The mean (s.d.) score of SIS-8 was 6.88 (2.90).

One hundred forty-four suicides (59.5%) had sought for help in the last month prior to death. Most commonly utilized health service was physicians other than psychiatrists (103, 42.6%), followed by village doctors (55, 22.7%), traditional Chinese medicine practitioners (nine, 3.7%), and mental health professionals (eight, 3.3%).

### Diagnosis of mental disorders

As shown in Table 2, 122 (50.4%) suicides and 12 (5.0%) comparisons met at least one diagnosis of mental disorder. The most common mental disorder in the suicide group was mood disorders (120, 42.1%), followed by psychotic disorders (15, 6.2%), substance use disorder (14, 5.8%), and anxiety disorders (seven, 2.9%). Sixteen suicides and one comparison had two diagnoses. The prevalence of any mental disorder, mood disorders, psychotic disorders, and alcohol dependence was significantly higher in suicide than those in comparisons, while the difference of anxiety disorders did not reach statistical significance.

**Table 2.** Diagnosis of DSM-IV mental disorders among 242 completed suicide and 242 living comparisons

Mental disorders	Completed suicides	Living comparisons	χ^2^	*p*
Mood disorders	102 (42.1%)	8 (3.3%)	103.95	<0.001
Major depressive disorder	82 (33.9%)	6 (2.5%)		
Major depressive episode	14 (5.8%)	0		
Bipolar disorder	5 (2.1%)	0		
Dysthymia	1 (0.4%)	2 (0.8%)		
Psychotic disorders	15 (6.2%)	1 (0.4%)	12.67	<0.001
Schizophrenia	3 (1.2%)	1 (0.4%)		
Psychotic disorders due to general medical condition or substance use	3 (1.2%)	0		
Psychotic disorders not otherwise specified	9 (3.7%)	0		
Alcohol dependence	14 (5.8%)	2 (0.8%)	9.308	0.002
Anxiety disorders	7 (2.9%)	2 (0.8%)	–	0.176^a^
Phobic disorder	3 (1.2%)	2 (0.8%)		
Panic disorder	2 (0.8%)	0		
Post-traumatic stress disorder	2 (0.8%)	0		
Any DSM-IV Axis I diagnosis	122 (50.4%)	12 (5.0%)	124.87	<0.001

a Fisher's exact χ^2^ test.

### Risks of elderly suicide: multivariable regression

Conditioned multivariable logistic regression was used to determine the risk factors of suicide. Independent variables included in the model were marital status, employment, living alone, being left-behind, social support, stressful life events, depressive symptoms (GDS score was entered as a continuous variable), and diagnosis of mental disorder. Four variables entered the final model: unstable marital status (OR 4.19, 95% CI 1.61–10.92), unemployed (compared with employed, OR 4.43, 95% CI 1.09–17.95), depressive symptoms (OR 1.34, 95% CI 1.21–1.48), and mental disorder (OR 6.28, 95% CI 1.75–22.54) (Table 3).

**Table 3.** Risks of completed suicide among rural elderly in China

Independent variables	*p*	OR (95% CI)
Marital status		
Stable marital status		1 (ref)
Unstable marital status	0.003	4.19 (1.61–10.92)
Employment		
Employed		1 (ref)
Unemployed	0.037	4.43 (1.09–17.95)
Retired	0.549	2.04 (0.20–20.76)
Depressive symptom (GDS score)	<0.001	1.34 (1.21–1.48)
Mental disorder		
No diagnosis		1 (ref)
Any diagnosis of mental disorder	0.005	6.28 (1.75–22.54)

Backward stepwise (likelihood ratio) method was used in this conditioned logistic regression model. Overall (score) χ^2^ = 174.751, *p* < 0.001.

### Test of the structural equation model

**Fig. 1.**
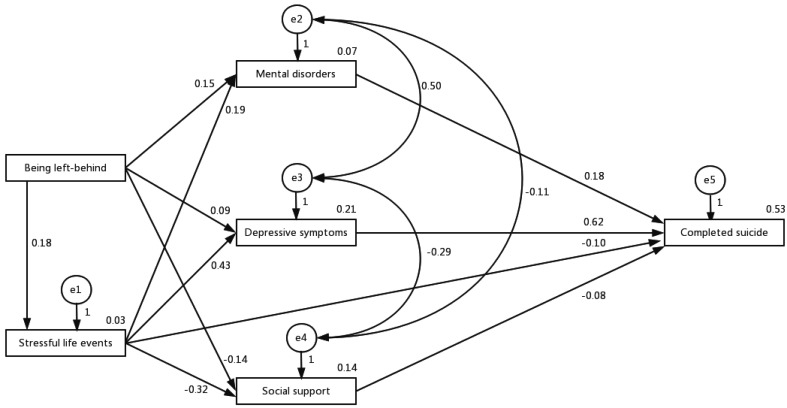
The relationship between being left-behind and suicide mediated by life events, social support, depressive symptoms, and mental disorder. The mediating effects of life events, social support, depressive symptoms, and mental disorder on the relationship between being left-behind and suicide were examined using structural equation model. χ^2^ (1) = 1.717, *p* = 0.190, GFI = 0.999, AGFI = 0.975, TLI = 0.986, and RMSEA = 0.039.

Verification analysis of the structural equation model proved that the theoretical model was well validated (Fig. 1). The χ^2^ test showed a significant probability >0.05 [χ^2^ (1) = 1.717, *p* = 0.190]. GIF analysis showed that the model fit was high: GFI = 0.999, AGFI = 0.975, TLI = 0.986, and RMSEA = 0.039. The standardized total effects of predictors on suicide were: depressive symptom 0.615, life events 0.230, mental disorder 0.179, being left-behind 0.136, social support −0.085.

## Discussion

Using a strict definition, being left-behind was significantly more prevalent in elderly suicides than in living comparisons (17% *v.* 10%). Structural equation model showed that being left-behind could elevate the suicide risk through increasing life stresses, depressive symptoms, mental disorder, and decreasing social support. This may explain our result that after controlling for these mediating factors using logistic regression, being left-behind is no longer associated with completed suicide. Emotional and instrumental support from family members, particularly adult children, is the central provider of care for rural elderly because of the deficiency of external support (He and Ye, 2014). Although a study in Thailand showed that parents with children outmigrated received more economic remittance and therefore had less depressive symptoms (Abas *et al.* 2009), it seems that this is not the case in China. Migrants are generally cheap labor in cities and have limited income. Studies at population level found that urbanization contributed to the decrease of suicide rates in general population (Sha *et al.* 2017); however, this study indicates that urbanization may increase elderly suicide risk at the individual level. Suicide prevention strategies should be developed for left-behind older adults in rural China, as well as in other developing and urbanizing countries.

The prevalence of mental disorders in elderly suicides was 50.4% (122/242), mostly mood disorders (42.1%, 102/242). These results were similar compared with previous studies in China. In a study of 304 suicides older than 55, authors reported that 64.8% suicides had at least one diagnosis of mental disorder, and the most common diagnosis was mood disorder (45.7%) (Zhou *et al.* 2004). Study in youth suicides in China showed that the prevalence of any DSM-IV mental disorder and depression was 48.0% and 34.9%, respectively (Zhang *et al.* 2010). The prevalence of mental disorder among elderly suicides in different countries varied from 47% to 96.5%, but depressive disorder was consistently the most common mental disorder (Waern *et al.* 2002; De Leo *et al.* 2013; Innamorati *et al.* 2014). Different methods used to detect and diagnose mental disorders across these studies may contribute to these differences. The absence of diagnosable mental disorder does not indicate that there is no psychological distress. Depressive symptoms are highly prevalent among elderly suicides: most suicides (94%) had at least moderate depressive symptoms, and nearly two-third had severe symptoms. Both mental disorder and depressive symptoms are independent risk factors for elderly suicide after controlling for other risks.

The most frequently used suicide means in our sample were pesticides, which is consistent with the previous studies in China (Phillips *et al.* 2002*b*; Zhang and Li, 2011). In earlier studies, in older (⩾55) (Wang *et al.* 2007) and youth suicides (Zhang and Li, 2011), pesticide was used in 52% and 66% suicides, while hanging was used in 28% and 10.5% suicides, respectively. These differences might reflect the decrease of availability of pesticides in the past decade in rural China: pesticides were stored at home in 75.3% youth suicides (Zhang and Li, 2011). Another possibility is that elderly suicides have stronger suicide intent and are more likely to use violent means such as hanging.

Our results have important implications in suicide prevention. Firstly, despite lower than previous studies, pesticide is still the most frequently used suicide means in rural China. Further efforts in restricting the accessibility of pesticides in rural areas are warranted. Secondly, nearly 60% elderly suicides had sought for help in the last month but only 3% had seen a mental health professional. This indicates that training rural physicians in identifying and referring individuals at suicide risk may prevent suicide among rural elderly. Thirdly, mental disorders and depression are important risk factors for suicide among elderly, and these conditions are rarely recognized or treated. To treat mental disorders, particularly depression, is of key importance for the prevention of suicide among elderly. Fourthly, we may buffer the negative impact of being left-behind through providing social support and enhancing social connections, treating mental disorder including depression, and mitigating life stress.

Several limitations need to be considered when interpreting the results. Firstly, because of the lack of comprehensive vital reporting system in China, we relied on local public health professionals to identify suicide cases. Misclassification and under-report of suicide is a concern. We trained the personnel who were involved in reporting suicide death, and the manner of death was determined by trained interviewers. By doing so, we identified 242 suicide deaths and the male/female ratio in the sample was 1.26 (135/107), close to previously reported male/female ratio of 1.4 among Chinese elderly suicides in 2013–2014 (Zhong *et al.* 2016). Secondly, there are methodological limitations in psychological autopsy studies. The use of proxy informants, the lack of blinding about suicide and comparison, and interviewing informants of suicides 2–6 months after death may have an impact on the reliability of the data. Third, it is important to establish temporal relationship between being left-behind and the mediating factors that were examined in the structural equation model. Most mediating factors happened during the last 12 months before death or investigation, which means during the period of being left-behind. However, a few of them might have started before being left-behind, for example, persistent financial difficulties, chronic mental illnesses such as schizophrenia.

## References

[ref1] Abas MA, (2009) Rural-urban migration and depression in ageing family members left behind. British Journal of Psychiatry 195, 54–60. 19567897 10.1192/bjp.bp.108.056143 PMC2802522

[ref2] Chan AC (1996) Clinical validation of the Geriatric Depression Scale (GDS): Chinese version. Journal of Aging and Health 8, 238–253. 10160560 10.1177/089826439600800205

[ref3] Chiu HF, (2004) Elderly suicide in Hong Kong – a case-controlled psychological autopsy study. Acta Psychiatrica Scandinavica 109, 299–305. 15008804 10.1046/j.1600-0447.2003.00263.x

[ref4] Conwell Y, (1996) Relationships of age and axis I diagnoses in victims of completed suicide: a psychological autopsy study. American Journal of Psychiatry 153, 1001–1008. 8678167 10.1176/ajp.153.8.1001

[ref5] De Leo D, (2013) Suicides in older adults: a case-control psychological autopsy study in Australia. Journal of Psychiatric Research 47, 980–988. 23522934 10.1016/j.jpsychires.2013.02.009

[ref6] He CZ and Ye JZ (2014) Lonely sunsets: impacts of rural–urban migration on the left-behind elderly in rural China. Population, Space and Place 20, 352–369.

[ref7] He XY, Xiao SY and Zhang DX (2008) Reliability and validity of the Chinese version of geriatric depression scale: a study in a population of Chinese rural community-dwelling elderly. Chinese Journal of Clinical Psychology 16, 473–475.

[ref8] Innamorati M, (2014) Suicide in the old elderly: results from one Italian county. The American Journal of Geriatric Psychiatry 22, 1158–1167. 23890752 10.1016/j.jagp.2013.03.003

[ref9] Knodel J and Saengtienchai C (2007) Rural parents with urban children: social and economic implications of migration for the rural elderly in Thailand. Population, Space and Place 13, 193–210.

[ref10] Kreager P (2006) Migration, social structure and old-age support networks: a comparison of three Indonesian communities. Aging and Society 26, 37–60. 10.1017/S0144686X05004411 PMC3672838 23750063

[ref11] Li X, Xiao Z and Xiao S (2009) Suicide among the elderly in mainland China. Psychogeriatrics 9, 62–66. 19604327 10.1111/j.1479-8301.2009.00269.x

[ref12] Liu S, (2015) Spatiotemporal variation and social determinants of suicide in China, 2006–2012: findings from a nationally representative mortality surveillance system. Psychological Medicine 45, 3259–3268. 26138093 10.1017/S0033291715001269

[ref13] National Bureau of Statistics of China (2017) China Statistical Yearbook *(*2016*)*. Beijing, China: China Statistics Press.

[ref14] Phillips MR, Li X and Zhang Y (2002 *a*) Suicide rates in China, 1995–99. The Lancet 359, 835–840. 10.1016/S0140-6736(02)07954-0 11897283

[ref15] Phillips MR, (2002 *b*) Risk factors for suicide in China: a national case-control psychological autopsy study. The Lancet 360, 1728–1736. 10.1016/S0140-6736(02)11681-3 12480425

[ref16] Rubenowitz E, (2001) Life events and psychosocial factors in elderly suicides – a case-control study. Psychological Medicine 31, 1193–1202. 11681545 10.1017/s0033291701004457

[ref17] Sha F, Yip PS and Law YW (2017) Decomposing change in China's suicide rate, 1990–2010: ageing and urbanisation. Injury Prevention 23, 40–45. 27312962 10.1136/injuryprev-2016-042006

[ref18] Waern M, (2002) Mental disorder in elderly suicides: a case-control study. American Journal of Psychiatry 159, 450–455. 11870010 10.1176/appi.ajp.159.3.450

[ref19] Wang CW, Chan CL and Yip PS (2014) Suicide rates in China from 2002 to 2011: an update. Social Psychiatry and Psychiatric Epidemiology 49, 929–941. 24240568 10.1007/s00127-013-0789-5

[ref20] Wang SF, (2007) The relationship between physical disease and depression in 304 elderly suicides. Chinese Journal of Gerontology 27, 2212–2214.

[ref21] Wang Z, (2013) Anxiety disorders and its risk factors among the Sichuan empty-nest older adults: a cross-sectional study. Archives of Gerontology and Geriatrics 56, 298–302. 23022057 10.1016/j.archger.2012.08.016

[ref22] Wang G, (2017) Loneliness and depression among rural empty-nest elderly adults in Liuyang, China: a cross-sectional study. BMJ Open 7, e016091. 10.1136/bmjopen-2017-016091 PMC5639988 28988166

[ref23] Xiao L and Xu HL (2008) The development of life events scale for the elderly. Chinese Journal of Behavioral Medical Science 17, 182–184.

[ref24] Yang G, (2013) Rapid health transition in China, 1990–2010: findings from the Global Burden of Disease Study 2010. The Lancet 381, 1987–2015. 10.1016/S0140-6736(13)61097-1 PMC7159289 23746901

[ref25] Zhang J and Li Z (2011) Suicide means used by Chinese rural youths: a comparison between those with and without mental disorders. Journal of Nervous and Mental Disease 199, 410–415. 21629021 10.1097/NMD.0b013e31821d3ac7 PMC3205915

[ref26] Zhang J, Xiao S and Zhou L (2010) Mental disorders and suicide among young rural Chinese: a case-control psychological autopsy study. American Journal of Psychiatry 167, 773–781. 20395398 10.1176/appi.ajp.2010.09101476 PMC3210861

[ref27] Zhang J, (2003) Studying Chinese suicide with proxy-based data: reliability and validity of the methodology and instruments in China. The Journal of Nervous and Mental Disease 191, 450–457. 12891092 10.1097/01.NMD.0000081613.03157.D9 PMC2758605

[ref28] Zhang J, (2014) The change in suicide rates between 2002 and 2011 in China. Suicide and Life-Threatening Behavior 44, 560–568. 24690079 10.1111/sltb.12090

[ref29] Zhang D, (2017 *a*) Characteristics of the Chinese rural elderly living in nursing homes who have suicidal ideation: a multiple regression model. Geriatric Nursing 38, 423–430. 28347559 10.1016/j.gerinurse.2017.02.005

[ref30] Zhang W, (2017 *b*) Prevalence and risk factors for attempted suicide in the elderly: a cross-sectional study in Shanghai, China. International Psychogeriatrics 29, 709–715. 27998320 10.1017/S1041610216002283

[ref31] Zhong BL, Chiu HF and Conwell Y (2016) Rates and characteristics of elderly suicide in China, 2013–14. Journal of Affective Disorders 206, 273–279. 27639861 10.1016/j.jad.2016.09.003

[ref32] Zhou MG, (2004) Analysis of negative life events among 304 elderly suicide victims. Zhonghua Liu Xing Bing Xue Za Zhi 25, 292–295. 15231194

